# Relationship between left ventricular cavity size and transient ischaemic dilation ratio on dipyridamole stress single-photon emission computerized tomography myocardial perfusion imaging in a female Asian population

**DOI:** 10.1093/ehjimp/qyaf102

**Published:** 2025-08-06

**Authors:** Chun Hui Sharmaine Wong, Min Sen Yew

**Affiliations:** Department of Cardiology, Tan Tock Seng Hospital, 11 Jalan Tan Tock Seng, Singapore 308433, Singapore; Department of Cardiology, Tan Tock Seng Hospital, 11 Jalan Tan Tock Seng, Singapore 308433, Singapore

**Keywords:** myocardial perfusion imaging (MPI), transient ischaemic dilation (TID), TID ratio (TIDr), left ventricular cavity size

## Abstract

**Aims:**

Single-photon emission computerized tomography (SPECT) myocardial perfusion imaging (MPI) has reduced accuracy in patients with small left ventricular (LV) size. Although transient ischaemic dilation (TID) is a sign of extensive coronary artery disease when accompanied by perfusion defects, its significance with normal perfusion remains unclear. We aim to study the relationship between the LV size and the TID ratio (TIDr) amongst females with normal SPECT MPI.

**Methods and results:**

Retrospective single-centre study of female patients with normal dipyridamole stress MPI, defined as the summed stress score = 0 with both stress and rest LV ejection fraction ≥50% on gated images. Small LV was defined as a gated rest end diastolic volume (EDV) below the 20th percentile of the study cohort. TIDr was derived using the quantitative perfusion SPECT software. There were 107 female patients (mean age—70) included. The threshold for small LV size was determined to be an EDV of <36.6 mL. Patients with or without small LV were similar in age, ethnicity, body mass index, and comorbidities. TIDr was significantly greater for patients with small LV (1.33 vs. 1.28, *P* = 0.042). There was a significant negative correlation between the resting EDV and the TIDr (*r* = −0.34, *P* < 0.001), which remained significant after controlling for age, body mass index, resting left ventricular ejection fraction, diabetes mellitus, and hypertension (*r* = −0.35, *P* < 0.001).

**Conclusion:**

In females with a normal dipyridamole stress SPECT MPI, TIDr is significantly higher in those with small LV. LV size should be considered when interpreting TID in females with otherwise normal MPI.

## Graphical Abstract

**Graphical Abstract:**
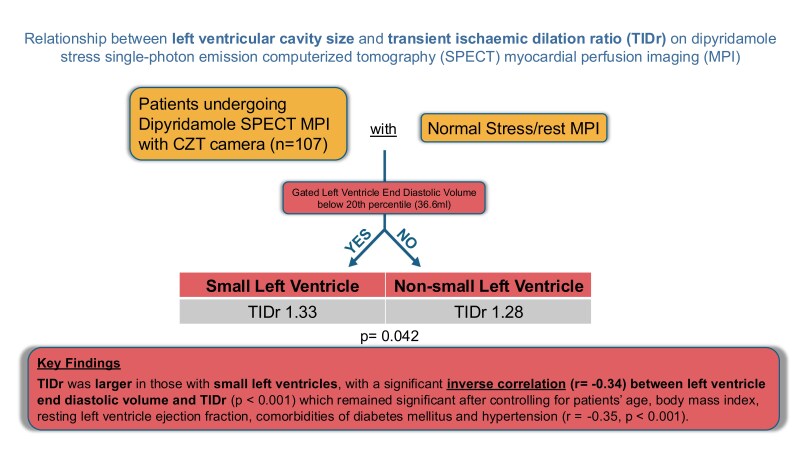

## Introduction

Stress single-photon emission computerized tomography (SPECT) myocardial perfusion imaging (MPI) is widely used for the evaluation of suspected coronary artery disease (CAD) and provides information regarding perfusion and function. SPECT MPI can also identify high-risk non-perfusion markers of ischaemia, such as post-stress transient ischaemic dilation (TID) of the left ventricle (LV). TID is believed to be a result of globally reduced tracer uptake from subendocardial ischaemia that gives the appearance of LV dilation post-stress.^1^ Abnormal TID ratio (TIDr) occurring with perfusion defects may signify extensive multivessel CAD and is strongly associated with the increase in major adverse cardiac events (MACE).^2–5^ The significance of TID occurring in MPI with no perfusion defects is more controversial but may identify high-risk patients with balanced ischaemia or globally reduced myocardial flow reserve.

Commercially available post-processing software can quantify the TIDr by comparing the endocardial LV volumes measured in ungated post-stress and rest short-axis images. However, TIDr thresholds vary and can be affected by factors including gender and LV cavity size.^6–8^ In certain populations, such as females of Asian ethnicity, where the prevalence of small LV cavity size is substantial, interpretation of TIDr may be affected. In this study, we aim to investigate the relationship between the LV cavity size and the TIDr in a female Asian population with otherwise normal SPECT MPI.

## Methods

### Study population

A single-centre retrospective observational study was conducted for patients with normal dipyridamole stress/rest technetium-99m (Tc-99m) tetrofosmin SPECT MPI in our institution between 1 January 2014 and 31 December 2015. We gathered data on demographics, body mass index (BMI), and medical comorbidities from electronic medical records. Normal SPECT MPI was defined as a summed stress score of 0 and both rest and post-stress LV ejection fraction (LVEF) of at least 50%. Patients with known CAD were excluded.

**Figure 1:**
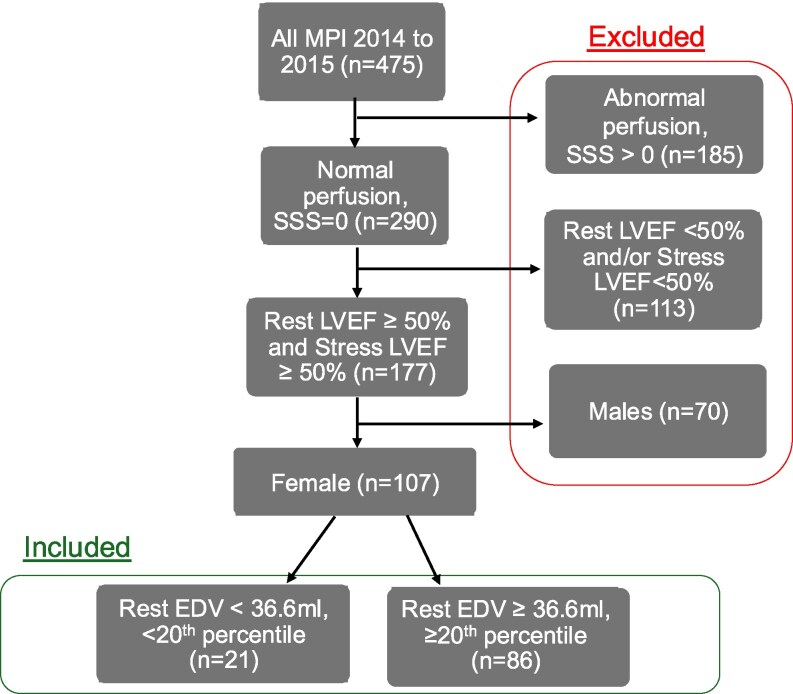
Flowchart of inclusion and exclusion of participants in the study.

A subgroup of patients with small LV cavity, as defined by gated rest LV end diastolic volume (EDV) below the 20th percentile of the overall study population, was identified and compared against those with non-small LV cavity. LV EDV was selected as the primary measure of LV size to maintain alignment with the majority of prior studies and current consensus in cardiology imaging practice.^9^ EDV also remains the preferred standard for defining LV chamber size, as it is less affected by transient changes in contractility or afterload. Due to the relatively fewer male patients undergoing pharmacological stress, with normal MPI and small LV cavity size, only the results of female patients will be reported here (*Figure 1*).

### Imaging procedure

All patients were scanned with a single cadmium zinc telluride gamma camera (Discovery NM 530c, GE Healthcare). All patients underwent pharmacological stress MPI with intravenous dipyridamole. For the one-day protocol, 8 mCi of Tc-99m tetrofosmin was used for rest imaging and 24 mCi for stress imaging. For most patients on a two-day protocol, 20 mCi was administered for each imaging session. Patients with body weight exceeding 80 kg underwent a two-day protocol with 25–30 mCi of Tc-99m tetrofosmin for each imaging, depending on actual body weight. Pre-test preparation, cardiac stress testing, and image acquisition and processing were performed in accordance with standard published protocols.^10,11^ Post-stress prone imaging was routinely performed unless not tolerated by the patient. Gated images were acquired post-stress and rest in the supine position by dividing the cardiac cycle into eight frames.

### Image interpretation

MPI images were processed on a dedicated workstation (Xeleris, GE Healthcare). An experienced nuclear cardiologist reviewed all images for the complete absence of perfusion defects. TIDr was automatically generated using the quantitative perfusion SPECT (Cedars-Sinai) software, based on the ratio of non-gated endocardial LV volumes in short-axis images post-stress and rest. Gated data were reviewed using the quantitative gated SPECT (Cedars-Sinai) software. Manual correction of contours was made by the nuclear cardiologist whenever needed.

### Statistical analysis

Statistical analysis was performed using IBM SPSS Statistics software version 16.0; significance tests were two-sided at the 5% significance level. Continuous variables were presented as mean with standard deviation if normally distributed. Differences between continuous variables were compared using a two-sample *t*-test. Categorical variables were presented as frequencies and percentages. Differences for categorical variables were compared with the χ^2^ test. A two-tailed *P*-value of <0.05 was considered statistically significant. The Pearson correlation analysis was used to compare correlations between continuous variables. Partial correlation analyses were conducted to examine the association between the TIDr and the EDV while adjusting for potential confounding variables, including age, BMI, rest LVEF, diabetes mellitus, and hypertension.

## Results

### Baseline characteristics

A total of 107 female patients met the inclusion criteria and were included for analysis. The mean age was 70 years, and the mean gated rest EDV was 50.2 mL. The threshold for a small LV cavity, as defined by the gated LV EDV below the lowest pentile of the overall population, was calculated to be 36.6 mL. Patients with or without small LV cavity were similar in terms of ethnicity, BMI, and prevalence of cardiovascular risk factors, such as diabetes (56.1% of the total population), hypertension (83.2%), and dyslipidaemia (80.4%). There was no statistically significant difference with respect to the protocol (one day vs. two days) used between the compared groups. This is summarized in *Table 1*.

**Table 1:** Comparison of patient groups with small and non-small LV

	All	Small LV (EDV < 20th percentile)	Non-small LV (EDV ≥ 20th percentile)	*P* value
Participants (%)	107	21 (19.6%)	86 (80.4%)	
Age (years)	68.7 ± 10.6	73.4 ± 11.4	69.1 ± 10.7	0.109
Ethnicity (%)				0.701
Chinese	78 (72.9%)	15 (71.4%)	63 (73.3%)	
Malay	14 (13.1%)	3 (14.3%)	11 (12.8%)	
Indian	11 (10.3%)	3 (14.3%)	8 (9.3%)	
Others	4 (3.7%)	0 (0%)	4 (4.7%)	
BMI (kg/m^2^)	27.1 ± 9.9	26.0 ± 5.9	28.0 ± 6.2	0.202
Diabetes mellitus (%)	60 (56.1%)	13 (61.9%)	47 (54.7%)	0.548
Hypertension (%)	89 (83.2%)	16 (76.2%)	73 (84.9%)	0.340
Dyslipidaemia (%)	86 (80.4%)	17 (81.0%)	69 (80.2%)	0.941
Protocol (%)				0.783
One-day	69 (64.5%)	13 (61.9%)	56 (65.1%)	
Two-day	38 (35.5%)	8 (38.1%)	30 (34.9%)	
Rest LV EDV (mL)	50.2 ± 17.0	28.3 ± 7.9	55.5 ± 14.1	**<0**.**001**
Rest LVEF (%)	77.4 ± 8.4	86.7 ± 6.3	75.1 ± 7.3	**<0**.**001**
Stress LVEF (%)	72.0 ± 9.6	84.1 ± 5.7	69.1 ± 7.9	**<0**.**001**
TIDr	1.29 ± 0.08	1.33 ± 0.11	1.28 ± 0.07	**0**.**042**

Values are presented as mean ± standard deviation or *n* (%).

Bold values indicate the statistically significant *P*-values less than 0.05.

### Interaction of cardiac size with TIDr

**Figure 2:**
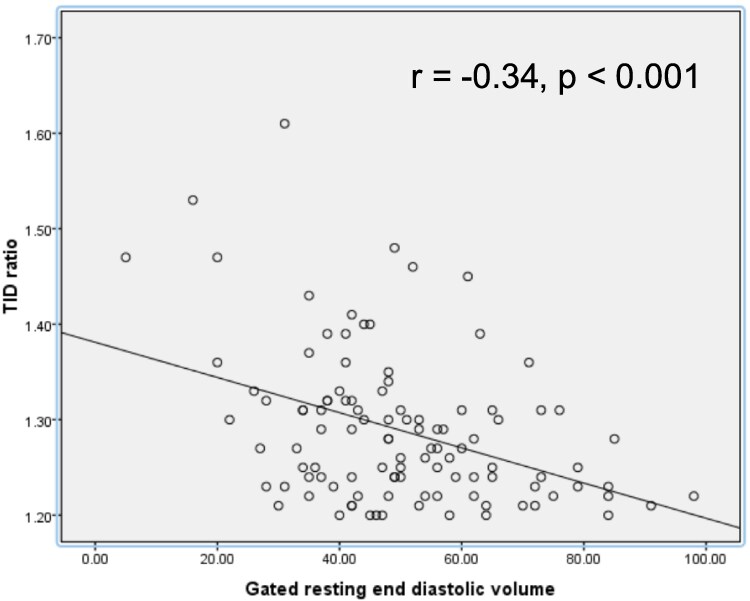
Linear regression analysis of TID ratio vs. gated resting end diastolic volume.

The mean TIDr was significantly greater for patients with smaller LV cavity size compared to patients with non-small LV cavity size (1.33 ± 0.11 vs. 1.28 ± 0.07, *P* = 0.042). There is a significant negative correlation between the rest EDV and the TIDr (r = −0.34, *P* < 0.001) by Pearson’s correlation analysis. The correlation between the rest EDV and the TIDr remained significant after controlling for age, BMI, resting LVEF, diabetes mellitus, and hypertension using the partial correlation analysis (*r* = −0.35, *P* < 0.001). This is summarized in *Figure 2*.

## Discussion

In this single-centre retrospective study of Asian females with no known CAD and a normal dipyridamole stress/rest SPECT MPI, we found that patients whose LV EDV fell below the lowest pentile had a significantly greater mean TIDr when compared to those with larger LV EDV. Findings are specifically applicable to female populations, given the exclusive inclusion of female participants in this study.

**Figure 3:**
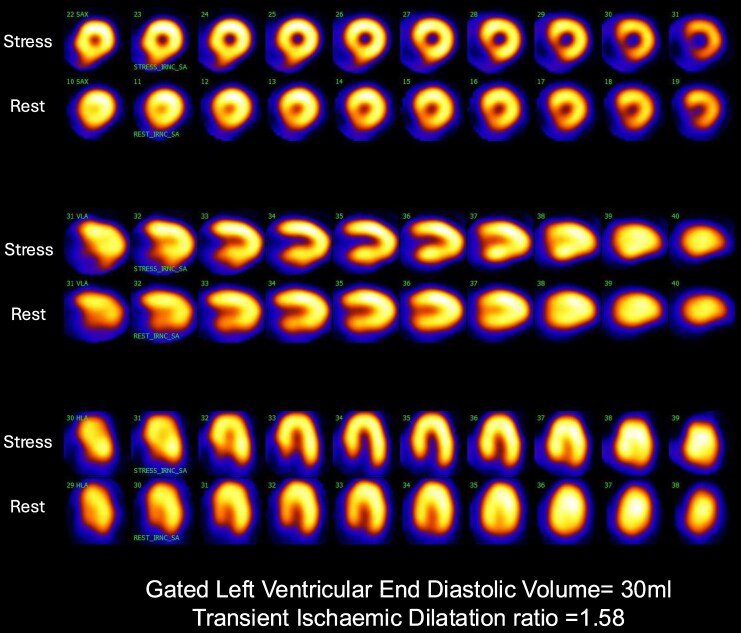
Representative non-gated dipyridamole stress and rest tomographic myocardial perfusion images from a female patient with small LV cavity demonstrating TID and normal myocardial perfusion.

*Figure 3* is a representative example of a female patient with elevated TIDr, small LV cavity, and normal myocardial perfusion. The clinical significance of this finding remains uncertain. Some studies observed a poorer prognosis, particularly in diabetics or those with known CAD.^5,7,12^ However, other studies did not find any differences in the CAD severity, survivability, or increase in the MACE.^13,14^ This discrepancy may be due to the low prevalence of TID and measurement variances in TIDr amongst patients with normal MPI, reducing predictive accuracy.

A common method to define the threshold TIDr value for normality is using two standard deviations above the mean in a reference population with normal MPI.^15^ Reported TIDr threshold values vary from 1.18 to 1.39,^7,16,17^ likely due to factors, such as heart rate,^18,19^ hypertensive response,^20^ LV hypertrophy (LVH),^21,22^ and patient positioning.^23,24^ Imaging protocols, including camera type, radiotracers, protocol duration, and stress modality also contribute.^25–28^

Population-specific factors, such as gender, affect TIDr. Females generally have higher mean TIDr than males, possibly due to their smaller LV cavities.^6,7^ Females consistently have smaller LV cavities across various imaging modalities, even after adjusting for body surface area.^29–32^ Furthermore, it has been shown that adjusting LV volumes for gender may improve the predictive accuracy of TIDr.^33^ This is particularly relevant in Asians, who have smaller LV cavity sizes compared to Caucasians and Blacks,^29,31^ especially among East Asian women.^34–36^ For example, Singaporean Chinese had smaller cardiac volumes compared to Caucasians on MRI.^37^

Small LV cavity size has been shown to impair MPI accuracy. In a study based on the REFINE SPECT registry, which includes predominantly Caucasians, patients within the lowest gender specific EDV pentile had significantly reduced area under the curve, sensitivity, specificity, and negative predictive value for MPI,^38^ with an EDV cut-off of 48.6 mL, compared to the lower value of 36.6 mL in our study cohort.

The impact of LV cavity size on TIDr remains underexplored. A single-centre study using a general-purpose gamma camera found an inverse relationship between the TIDr and the LV volume, with the TIDr increasing from 1.20 to 1.37 between the highest and lowest deciles of rest ungated LV volume.^8^ Overestimation of TIDr in small LV cavities may be influenced by partial volume effects, wherein limited spatial resolution causes underestimation of endocardial counts during stress imaging structures.^13,22^ However, this may not fully explain the findings in our study, as we had utilized a cadmium zinc telluride camera with higher spatial resolution compared to conventional cameras. Similarly, the REFINE SPECT registry data^38^ were obtained using solid-state scanners and were comparable to results from older studies using conventional Anger cameras, which demonstrated lower sensitivity, particularly in patients with smaller LV cavity size.^39,40^

LV hypertrophy (LVH), associated with small LV cavity size, is an independent predictor of TID and could confound the assessment of TIDr.^21,22^ While LVH was not assessed in our cohort, baseline systemic hypertension, a major contributor to LVH, was prevalent in the majority (83.2%) of patients, and was equally distributed between both groups.

While the generalizability and applicability of this study may be limited to female patients, we focused on females for several reasons. First, sex is known to affect TIDr measurements due to known physiological and anatomical differences.^6,35^ Given the multiple factors that can confound TIDr calculation, we wanted to focus only on female patients undergoing dipyridamole stress in this report to reduce heterogeneity in the study population to obtain clinically meaningful TIDr estimates. Second, Asian women, who typically have smaller LV cavities than their Caucasian counterparts, were underrepresented in earlier studies on TIDr, such as the REFINE SPECT registry.^38^ As such, more data specific to this group are needed to better inform clinical interpretation and decision-making. Finally, we also had insufficient male patients with a normal vasodilator stress MPI in our centre for meaningful statistical analysis. This is consistent with other studies showing that women are more likely to undergo pharmacological stress MPI than men,^41,42^ partly due to older age at CAD presentation.^30,43^ Also, men are more likely to be physically fitter, and those able to exercise were preferentially referred for other functional tests, such as exercise stress electrocardiogram or stress echocardiography.

Our findings highlight the LV cavity size as a potential confounder to the interpretation of TIDr in Asian females. This suggests that there may not be a single-universal ‘normal’ TIDr threshold. The significant negative correlation between the TIDr and the gated LV EDV further implies that TIDr may be a continuum that is influenced by the LV cavity size. Collectively, our findings serve as a reminder that clinicians should interpret single TIDr in context, integrating clinical presentation and other diagnostic data to make a well-informed assessment.

## Limitations

Our study has several limitations. First, this is a retrospective single-centre study involving a select group of Asian female patients undergoing dipyridamole stress MPI, hence limiting the generalizability of results to other populations, particularly males, scan or stress protocols, gamma camera technology or even post-processing software. Similarly, the patient numbers are small due to the highly selected inclusion criteria.

Second, we did not assess the impact of heart rate at the time of rest/post-stress scanning. A large heart rate difference during the rest and post-stress acquisition can affect the TIDr.^44^ However, heart rate at the time of imaging is not routinely recorded in clinical practice and is also not mandated by standardized nuclear imaging reporting guidelines.^11,45^ Ideally, this should be evaluated in a future controlled prospective trial.

Lastly, consistent with prior publications, we identified ‘normal’ patients based on the perfusion, gated LVEF, and absence of known CAD. Due to the retrospective nature of our study, coronary anatomical data were not available for most patients with ‘normal’ MPI did not undergo further invasive investigations. As such, ‘balanced ischaemia’ could not be confidently excluded in these patients. Similarly, we could not obtain reliable data on the MACE as many patients were discharged from follow-up after a normal MPI.

## Conclusion

In summary, there is a significant inverse correlation between the gated LV EDV and the TIDr amongst Asian females with otherwise normal dipyridamole stress SPECT MPI. Females with smaller LV cavities exhibited a higher mean TIDr compared to those with larger LV cavities. Further research involving larger populations, including males and other ethnic groups, is needed to validate these findings and explore their correlation with MACE.

## Data Availability

The data that support the findings of this study are not publicly available due to ethical restrictions but are available from the corresponding author upon reasonable request.

## Lead author biography

Dr Wong Chun Hui Sharmaine is a resident in the National Healthcare Group cardiology senior residency programme in Tan Tock Seng Hospital, Singapore. Her clinical interests include cardiac imaging.
